# Early Neolithic (ca. 5850-4500 cal BC) agricultural diffusion in the Western Mediterranean: An update of archaeobotanical data in SW France

**DOI:** 10.1371/journal.pone.0230731

**Published:** 2020-04-02

**Authors:** Laurent Bouby, Philippe Marinval, Frédérique Durand, Isabel Figueiral, François Briois, Michel Martzluff, Thomas Perrin, Nicolas Valdeyron, Jean Vaquer, Jean Guilaine, Claire Manen

**Affiliations:** 1 ISEM, CNRS, EPHE, IRD, Université Montpellier, Montpellier, France; 2 ASM, CNRS, MCC, Inrap, Université Paul Valéry, Montpellier, France; 3 TRACES, CNRS, EHESS, Inrap, MCC, Université Toulouse Jean Jaurès, Toulouse, France; 4 Inrap Grand Sud-Ouest, Bègles, France; 5 Inrap Méditerranée, KM Delta, Nîmes, France; 6 HNHP, UMR 7194, MNHN, Université de Perpignan, Sorbonne Universités, Perpignan, France; University at Buffalo - The State University of New York, UNITED STATES

## Abstract

Farming economy was first introduced to the coastal areas of Southern France by Impressa groups (ca. 5850–5650 cal BC), originating from Italy, and subsequently spread to the hinterland by Cardial/Epicardial communities (ca. 5400–4500 cal BC). Fruit and seed remains preserved in archaeological sites provide direct evidence of the botanical resources cultivated and collected by these ancient social groups. But the transition from hunter-gathering to agricultural subsistence strategies is still poorly known in the area, due to insufficient and sometimes outdated archaeobotanical studies. Here we present new results and a critical review of all the available archaeobotanical data, in order to characterize food plant resources, cultivation practices and their variations in time and space. The archaeological dataset is composed of 19 sites (20 site/phases) mostly located in the Mediterranean lowlands. Our results demonstrate that farming economy of the Impressa groups was focused on the cultivation of hulled wheats, with only slight differences compared to their South Italian origins. The contribution of naked cereals increased in the Cardial/Epicardial agriculture, in agreement with the situation in other areas of the Western Mediterranean. The subsistence economy of hinterland sites seems to include a wider contribution of wild fruits and more limited contribution of crops. However, the poor evidence of cultivation activities in the hinterland is likely due first to the difficulties to find and excavate the sites and perform large-scale archaeobotanical sampling. It is likely that agriculture played a significant but variable role between sites and territories.

## Introduction

Among all the characteristics of the European Neolithic (pottery making, polished axe blades, sedentism) the advent of food production economy, i.e. crop cultivation and animal husbandry, is regarded as especially prominent [1]. The transition from hunter-gathering to farming economic systems is considered a change with major consequences for the history of the world and of humanity. The exploitation of agricultural resources in the long run allowed human groups to rely on a range of more predictable resources than those from hunting and gathering and created the conditions for an unprecedented demographic growth. However, the Neolithisation of Europe is no longer interpreted as a steady and uniform diffusion, from East to West, of a new way of life but as a dynamic and arrhythmic process, opening the way to various strategies and economic situations, in balance with the environmental conditions [2]. The transition from hunter-gathering to farming economic systems was not necessarily an abrupt change. Because we have no means of measuring the quantities of food products produced and consumed by Neolithic people it is difficult to assess the contribution of agriculture to their subsistence and economy, as suggested by the ongoing debates about the respective contribution of wild resources and domesticated plants and animals, and about the variability of strategies in space and time [3, 4, 5, 6, 7].

In terms of the diffusion of the Neolithic, the western Mediterranean is globally homogeneous and related to the complex of Impressed Wares, whose origins are to be found in southern Italy. However, the chronology, the processes, the pathways and socio-economic contexts that led to this dissemination remain the subject of much debate [8, 9]. Within the “Impressed ware” cluster different cultural groups (Impressa, Cardial, Epicardial) are distinguished according to chronology, technical productions and economic resources [10].

The French Mediterranean coast was colonized at an early stage, around 5850 cal BC, by populations of Italian origin and belonging to the Impressa group [11]. Only a few Impressa sites are recognized and from what is known today it seems that the presence of these groups was restricted to the littoral area [11]. The links with the Italian Impressa complex are confirmed by the study of all cultural and technological aspects (ceramic and lithic systems, animal breeding, presence of obsidian from Palmarolla and Sardinia) [11, 12].

According to the available information, the Cardial complex develops at around 5400 cal BC in Languedoc while slightly earlier dates are reported in the Iberian Peninsula [12, 13, 14]. The Cardial origin is still under investigation. At around 5250 cal BC a cultural change takes place with the appearance, throughout a large area of southern France, of Epicardial groups (mainly defined on the basis of the ceramic decoration), which were contemporaneous with the Cardial ones for several centuries before fully replacing them [10]. With the Cardial, the Neolithic way of life experienced a major geographic expansion. Neolithic occupations progressively extended over a large part of the hinterland and Epicardial settlements even reached mountain areas. Various types of sites (open air, cave and rock-shelters) are encountered in diverse geographic settings [9]. The first Neolithic communities from the hinterland are unfortunately still poorly characterized, due to insufficient, often outdated excavations and to the difficulty of understanding stratigraphic contexts with atypical artifacts. In South-Western France, very little firm evidence of the presence of Mesolithic groups after 5800 cal BC is available. Based on current research, it is still difficult to identify any direct contacts between the first farming communities and the last hunters-gatherers [15].

Food plant resources and cultivation practices of the Early Neolithic groups are still poorly known and mostly documented by earlier studies in Southern France. The opportunities to engage in new large scale archaeobotanical investigations are very restricted due to the scarcity of archaeological surveys and new discoveries, even in the context of preventive archaeology. The few Early Neolithic sites recently found are often composed of scarce archaeological structures and cover a limited ground surface. In the open air settlements excavated, the use of mud architecture, the presence of scattered sherds and artifacts and the diversity of domestic structures remind us that these sites can often go undetected and that their number could therefore be underestimated [16]. Given the limited possibilities to initiate new investigations, the earliest archaeobotanical datasets should still be taken into account but bearing in mind the limits due to recovery methods and restricted sampling.

The aim of this paper is to examine the nature, chronology and development of farming activities. This work is based on investigations performed at new excavations and on a critical review of the currently available data, and was carried out as part of the “PROCOME–Continental extensions of Mediterranean Neolithisation” research program (ANR-13-CULT-0001-01). Our main objectives are: (1) to characterize the domestic and wild plant resources used by Early Neolithic groups for their subsistence and (2) to examine the spatio-temporal variations in cultivation activities in relation to cultural and environmental conditions.

## Material and methods

### 2.1. Data acquisition & archaeobotanical methods

**Fig 1:**
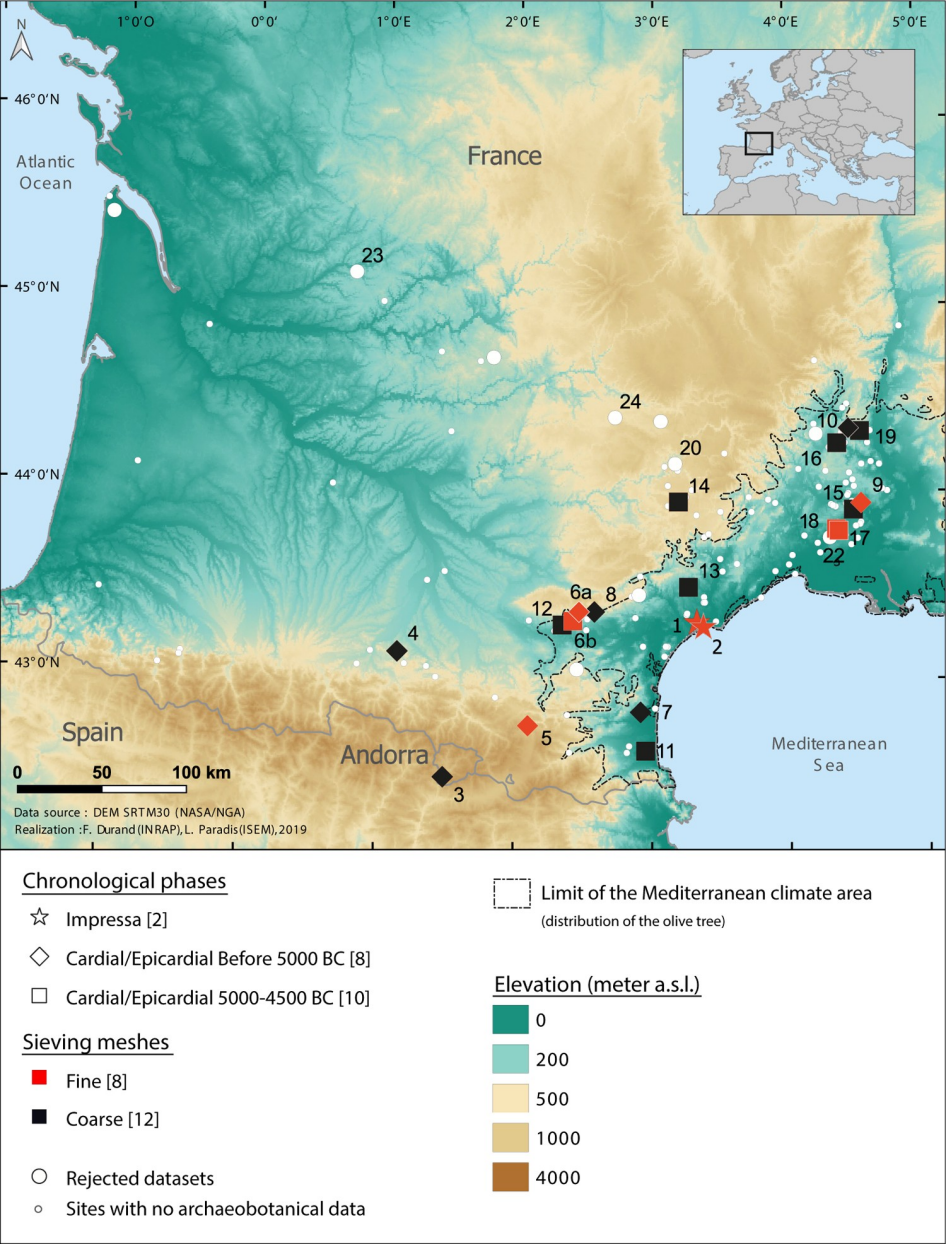
Map of Southwestern France showing the limits of distribution of the olive tree (Mediterranean bioclimatic area) and the location of the Early Neolithic sites, including those discussed in the paper which delivered archaeobotanical data. Between brackets: number of sites per category. Site identification numbers refer to Tables 1 and S1.

Our objective was to assemble all the archaeobotanical data that was available in order to document the Early Neolithic plant economy and progression of agriculture in south-western France, from the Mediterranean to the Atlantic coast (Fig 1). The geographical area considered is delimited by the Mediterranean Sea and the Pyrenees to the South, by the Rhone River to the East, the highlands of the Massif Central to the North and the Atlantic Ocean to the West. In order to gather as much data as possible and to ensure the validity of these data, we have implemented a two-fold approach, as described below. No permits were required for the described study, which complied with all relevant regulations. The samples analyzed are identified by site name and Stratigraphic Unit numbers (no specimen numbers). All the samples are stored in the repositories of ISEM (Montpellier), ASM (Montpellier), TRACES (Toulouse) and Inrap-Méditerranée (Béziers) and are accessible on demand.

**Table 1:** Site/phases that have been retained after critical revision of stratigraphic, cultural and radiocarbon data. In brackets: Department number.

Nb	Site/Occupation	Type	Radiocarbon	Samples and Nbr of dates^2^
			chronology^1^	
			cal BC (p = 95.4%)	
	**Impressa**			
1	Peiro Signado, Portiragnes (34)	Open air	5964–5721	2 SLS
2	Pont de Roque-Haute, Portiragnes (34)	Open air	6002–5638	5 SLS
	**Cardial/Epicardial (before ca 5000 cal BC)**			
3	Balma Margineda (Andorra)—C3, C3a, C3base	Cav/Shel	5662–4554	5 SLS
4	Abri Buholoup, Montberaud (31)—C3, C2	Cav/Shel	5221–4911	1 LLS
5	Roc de Dourgne, Fontanès de Sault (11)—C6	Cav/Shel	5485–5230	2 SLS
6a	Grotte Gazel, Sallèles Cabardès (11)—Gazel I-II	Cav/Shel	5462–4909	13 SLS
7	Cova de l'Esperit, Salses (66)—C2m, C2a	Cav/Shel	5210–5009	1 LSL
8	Baume Abeurador, Félines Minervois (34)—C2b	Cav/Shel	5466–5000	1 LLS
9	Tai, Remoulins (30) -	Cav/Shel	5367–4961	17 SLS
10	Baume d'Oullins, Le Garn (07)—C6—C5	Cav/Shel	5513–4848	18 SLS
	**Cardial/Epicardial (ca 5000–4500 cal BC)**			
11	Aspre del Paradis, Corneilla del Vercol (66)	Open air	5034–4691	2 SLS
12	Font Juvénal, Conques sur Orbiel (11)—C13, C12	Cav/Shel	5210–4545	5 SLS
6b	Grotte Gazel, Sallèles Cabardès (11)—Gazel III-IV	Cav/Shel	4897–4722	4 SLS
13	La Resclauze, Gabian (34)—C11	Open air	4837–4587	2 SLS
14	Abri Roc Troué, Ste Eulalie-Cernon (12)—St59	Cav/Shel	4931–4519	1 LSS
15	Baume Bourbon, Cabrières (30)—Sal2, eboulis	Cav/Shel	5484–4713	2 LLS
16	Grotte de l'Aigle, Mejannes le Clap (30)—C5	Cav/Shel	5214–4729	5 SLS
17	Mas de Vignoles 10, Nîmes (30)	Open air	5078–4792	1 SLS
18	Mas Neuf, Nîmes (30)—FS1007, US1231	Open air	5208–4844	3 SLS
19	Grotte St Marcel, St Marcel Ardèche (07) -Ck	Cav/Shel	5479–5064	1 LSL

Calibration with OxCal v 4.3.2 [20]; r:5 and IntCal13 atmospheric curve [21]

1. Range of all the dates available for the site/phases with calibration interval (95.4% probability) [22, 14]. In italics, poor reliability.

2. SLS = only short-lived samples; LLS = long-lived samples.

#### 2.1.1. New archaeobotanical analyses

Due to the shortage of Early Neolithic sites in Southwestern France, we tried in recent years to analyze sediment samples of any putative an Early Neolithic occupation, however limited. This led us to analyze samples from 6 different sites (S1 Table). The presence of Early Neolithic plant remains had previously been reported at two of these sites, Roquemissou [17] and Taï [18], while the other sites were investigated for the first time.

Except in Prairie du Lieu-Dieu, where a well with waterlogged sediments was investigated, sediments were processed by hand flotation, and the non-floating mineral residues systematically sieved. In all cases mesh sizes from 0.4 mm to 2 mm were used. At Prairie du Lieu-Dieu the samples were directly water sieved, using similar meshes. All the fractions were entirely sorted using a stereo-microscope and all plant remains identified using scientific reference literature and the comparison collections of modern seeds from the archaeobotany laboratories of ISEM (Montpellier) and TRACES (ArchéoScience plateform, Toulouse). Plant remains were systematically counted (Number of Plant remains—NPR) according to their anatomical and taxonomical origin, distinguishing entire specimens and fragments. The newly investigated samples are stored in the repositories of ISEM and TRACES.

#### 2.1.2. Data validation

All the available data, acquired recently or earlier, were critically appraised in order to be validated. The stratigraphic context of the archaeobotanical assemblages was systematically revised based on 1) resumption of fieldwork (new surveys, new stratigraphic observations), 2) new studies of the artifacts gathered from old excavations, 3) new radiocarbon dates including AMS dates on selected seeds from different sites. The radiocarbon samples were always composed of single seed remains, usually cereals (Cerealia, *Hordeum vulgare–*common barley, *Triticum aestivum/turgidum–*naked wheat, *Triticum dicoccum*—emmer and *Triticum* sp.–wheat), occasionally pulses (Fabaceae) and fruits (*Corylus avellana*—hazelnut, *Quercus* sp.—oak). Detailed information relative to the protocol of sample selection and AMS ^14^C dating procedure are published in [14].

Sampling information was recorded for each site, including number of samples, sieving methods, preservation of plant remains and number of recovered items. This is necessary to evaluate the representativity of the assemblages. To start with, sites were classified according to two levels of reliability, good when fine sieving was applied, moderate when only a coarse sieving was used (minimum mesh larger than 0.5 mm).

#### 2.1.3. Data processing and analysis

Before any computation or comparison between sites, the raw counts of plant remains were transformed as Minimum Number of Individuals using the formula: MNI = Number of Entire Seeds + ½ Number of Fragments.

The sites are grouped into three large chrono-cultural categories and the archaeobotanical data is recorded accordingly in sites with long chronologies: Impressa sites, Cardial/Epicardial sites dated before 5000 cal BC, Cardial/Epicardial sites after 5000 cal BC [9].

As sample size is highly variable from one site to another, sites are compared using percentage data rather than raw counts of plant remains. In order to better understand the effects of site characteristics on the overall changes in the archaeobotanical data we performed a correspondence factor analysis (CFA) and then explored using boxplots the distribution of the sites on the first two axes of the CFA according to chronological phase (Impressa, Cardial/Epicardial before 5000 cal BC, Cardial/Epicardial after 5000 cal BC), settlement type (open air *vs*. cave/rock shelter occupation) and recovery method (fine *vs*. coarse sieving). Only sites with MNI of at least 30 were used for the CFA and most of the figures. The CFA was performed on square-rooted transformed percentage data. Taxa which could not be identified precisely enough (Cerealia, *Triticum* sp.) were only considered as supplementary (passive) variables. These taxa were also excluded from the reference sums before calculating percentages.

#### 2.2. Description & representativeness of the dataset

Early studies and newly investigated sites amounted to a total of 30 sites representing 32 phases (1 site can be broken down into 2 phases). This amounts to about 18% of the known Early Neolithic sites in the area [9]. Data revision led to the exclusion of 11 of these sites as the stratigraphic context of the Early Neolithic occupation could not be ascertained in the light of new field and typo-chronological investigations, or the connection between the archaeobotanical assemblages and the Early Neolithic occupation was invalidated by radiocarbon dating.

Many of the eliminated sites correspond to old excavations and limited archaeobotanical studies, done with rough recovery techniques (Grotte de Chazelles, Abri Camprafaud, Abri Jean Cros, Roucadour and Lède du Gurp).

With the exception of Taï [19], archaeobotanical investigations on newly excavated sites bear limited results. In Combe Grèze, no significant results were obtained. In Prairie du Lieu-Dieu, the waterlogged assemblage from the well cannot be assigned with certainty to human activities rather than natural processes (no carbonized remains, no cultivated plants).

In the other sites (Le Cuzoul, La Farigoule, Roquemissou and Clos de Poujol), the results of radiocarbon dating of plant remains do not confirm the allocation of the archaeobotanical assemblages to the Early Neolithic. In general, the Early Neolithic occupations of these sites are very limited or documented by too restricted excavations, archaeological documentation and archaeobotanical sampling. On the other hand, the rather extensive sampling performed at Roquemissou failed up to now to provide any clear Early Neolithic archaeobotanical assemblage, while more than 3800 plant remains were retrieved for the Mesolithic and Late Neolithic layers. However, the Early Neolithic layers are still being excavated with ongoing archaeobotanical study.

**Fig 2:**
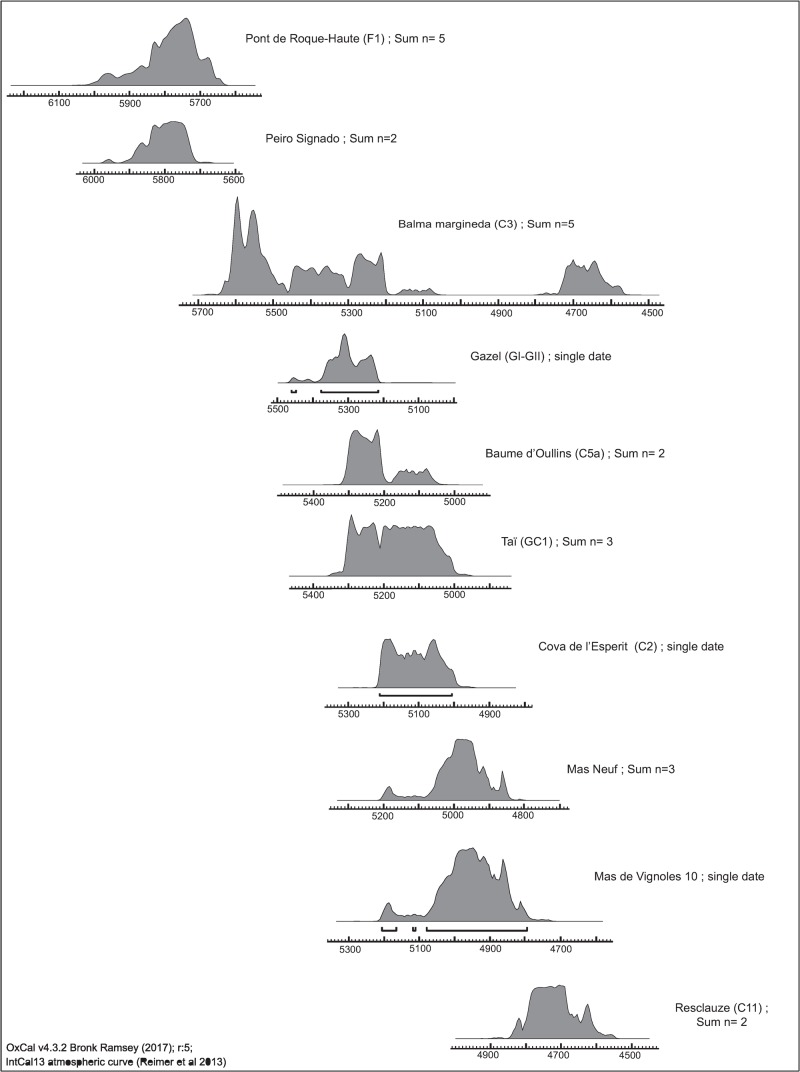
SMA radiocarbon dates performed on carbonized seeds from 10 sites (sum of probabilities when several dates at one site).

In short, only 19 sites and 20 site/phases can be retained (Tables 1 and S2). Radiocarbon dates performed on seeds confirm the expected chronology for 10 of these sites (Fig 2 and S3 Table). In the other 9 the stratigraphic context is firmly established and supported by several radiocarbon dates performed on other type of material (charcoals and bones). At Balma Margineda Early Neolithic artifacts and structures are observed in layer 3 (C3). Radiocarbon dates show that the site was occupied intermittently during the Early Neolithic, from about 5600 to 4500 cal BC [14]. However, due to the complexity of the stratigraphy and to post-depositional problems, the dates are randomly distributed in the Early Neolithic layer and it is not possible to subdivide the archaeobotanical assemblage in distinct chronological phases. Given that 4 of the 5 dates performed on seeds are earlier than 5000 cal BC we have decided to allocate the whole archaeobotanical assemblage to the category of the Cardial/Epicardial sites dated before 5000 cal BC. The opposite is the case at Grotte Saint Marcel. A single radiocarbon date on wood charcoal (MC 2376 6330 +- 90 BP; 5479–5064 cal BC) [23] is available. This result is not in agreement with the archaeological material, indicative of the recent phase of Cardial/Epicardial. Our hypothesis is that the radiocarbon date is affected by the well-known “old wood effect” and we decided to keep the site in the category Cardial/Epicardial post 5000 cal BC.

**Fig 3:**
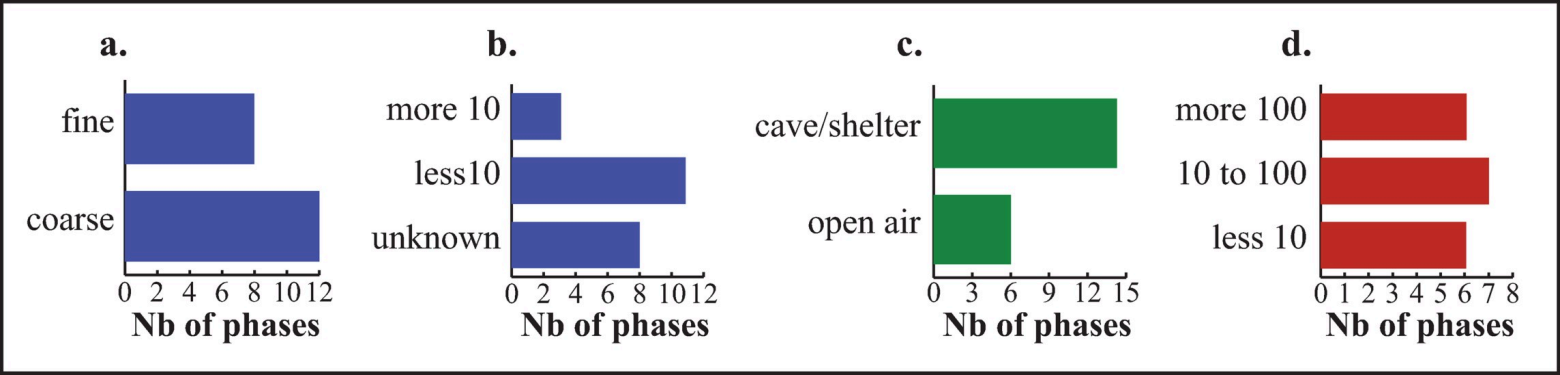
Characteristics of sampling carried out in Early Neolithic sites of South-Western France. (a) Sieving method: coarse (smallest mesh > 0.5 mm) vs. fine sieving (smallest mesh ≤ 0.5 mm), (b) Number of samples per site/phase, (c) Type of settlement, (d) Number of plant remains identified per phase (MNI).

**Fig 4:**
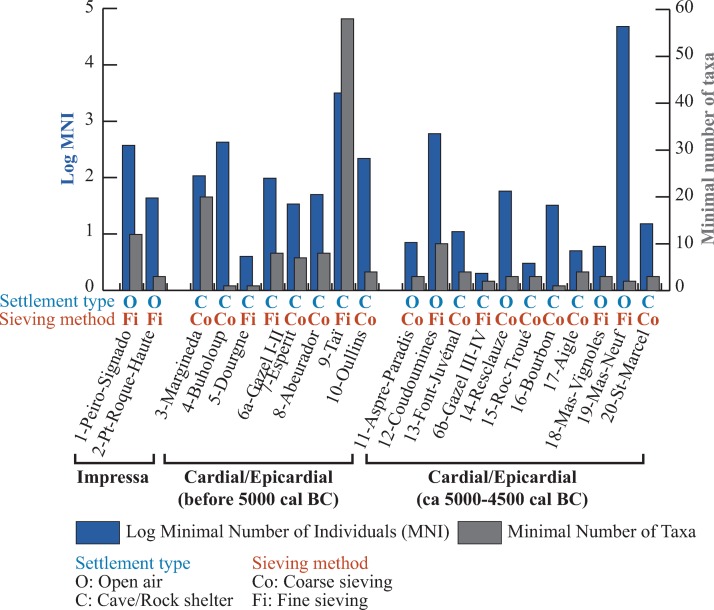
Number of plant remains and identified taxa in the sites (site/phases). The number of plant remains is expressed as the Log value of the MNI.

The final archaeobotanical dataset is largely limited to the Mediterranean region (Fig 1). This is the area where Early Neolithic sites are the most abundant, where the largest sites can be found, and consequently was the focus of most of the excavations and archaeobotanical studies. It is interesting to note that most of the archaeobotanical data that had to be discarded is from sites outside the Mediterranean region, including both recent and early investigations. This is clear evidence of how difficult it is to document the food plant economy of Early Neolithic populations in the temperate zone. Further data is available from caves/ rock shelters in the Mediterranean hinterland. Finally outside the Mediterranean bioclimatic zone we only have data from the cave and rock-shelter sites of Roc de Dourgne, Balma Margineda and Buholoup in the Pyrenean foothills, Font Juvenal and Grotte Gazel in the foothills of the Montagne noire and Roc Troué in the Grands Causses area (Fig 1). In the whole dataset the cave and rock-shelter sites prevail (Fig 3). In fact, the first phase of the Cardial/Epicardial (before 5000 cal BC) is only documented by cave and rock-shelter occupations (Fig 4). Only 6 open-air settlements are recorded, all located in the Mediterranean plain. They include the only two Impressa sites and Cardial/Epicardial sites after 5000 cal BC.

Sampling and recovery techniques are in many cases inadequate. The sites where only coarse sieving was carried out outnumber those where fine-sieved meshes were used. The number of archaeobotanical samples is often not specified in the old studies; only 3 site/phases are clearly reported to be documented by more than 10 samples. As a result of the poor sampling performed at many sites the number of identified plant remains is usually low; only about a third of the site/phases provided more than 100 plant remains. Taï, with 85 samples and more than 4000 plant remains, is by far the best investigated site [19]. A very large number of plant remains (MNI = 47450) was recovered at the site of Mas Neuf but they come from a single concentration of cereals found in a pit [24].

At all the sites only charred botanical remains were preserved. The minimal number of identified taxa is usually low (mean = 7.5; min = 1; max = 58) and is weakly correlated to the MNI (Spearman coefficient = 0.356, p-value = 0.123). It is no surprise that the highest numbers of identified taxa are found in the sites with the largest number of samples.

Except the case of Mas Neuf, and as far as we can judge with the limited information we have on the volumes of sampled sediments, all the sites provided open, low-density assemblages. In the sense of Jacomet et al. [25] open assemblages do not contain enough plant remains to be visible with the naked eye during excavations. They are usually the result of several episodes of deposition and contain material of mixed origins.

The final archaeobotanical dataset is limited and present inequalities between sites categories, especially between chronological phases. We should therefore be cautious when interpreting any trend observed in the data. In order to better evaluate their validity we compared our observations to the information available from Italy and Spain.

## 3. Results

### 3.1. Archaeobotanical data and variation in sample composition

**Fig 5:**
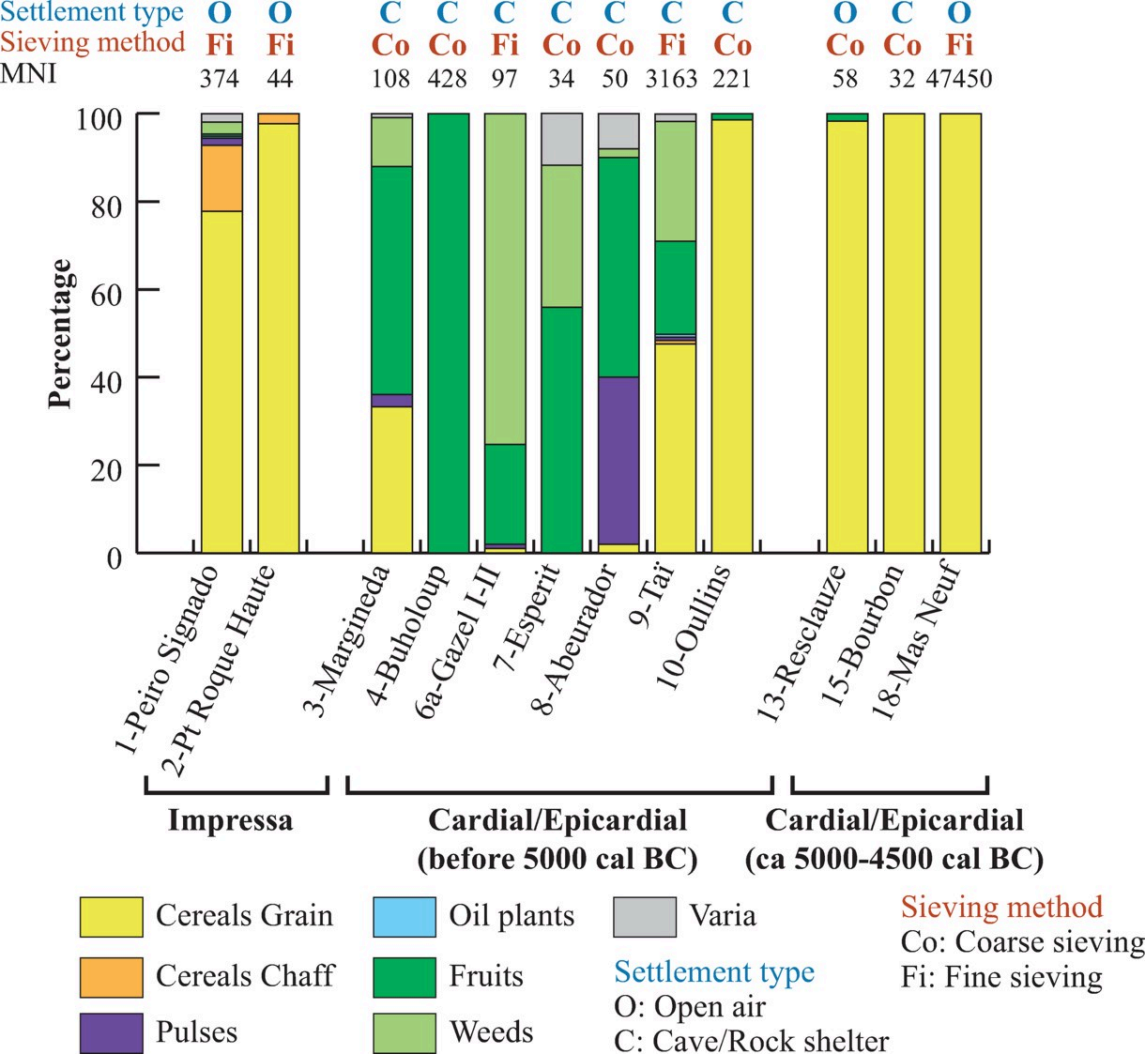
Proportions of the main categories of plants in the sites (site/phases) expressed as percentages calculated on the MNI. The MNI is indicated on the graph at the top of the bar of each site/phase. Only the site/phases with MNI ≥ 30 are considered in this graph.

A total of 52108 plant remains (MNI) and at least 64 taxa were identified in the 20 site/phases (S4 and S5 Tables). The majority of the archaeobotanical assemblages is dominated by crop remains (Fig 5).

**Fig 6:**
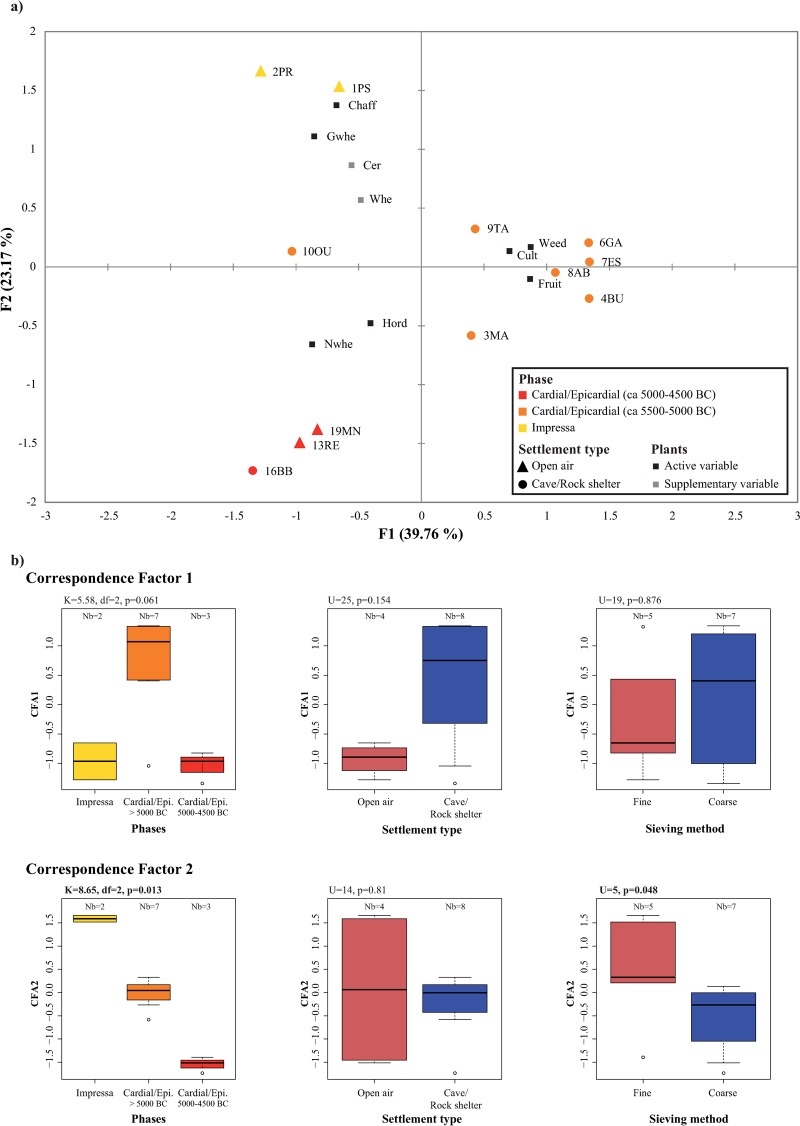
Correspondence factor analysis (CFA) performed on the abundance of plant remains (square-rooted transformed percentage data) in the sites (a) and visualization of the distribution of the sites on the first two axes according to characteristics associated to chronology, site type and sieving methods (b). Only site/phases with MNI ≥30 are considered. *Cerealia* and *Triticum* sp. are considered as supplementary data. The number of sites per group is indicated at the top of each boxplot. Statistical differences between groups of sites were tested using non parametric Mann-Whitney and Kruskal-Wallis tests. Results of the tests are indicated in bold on the graphs when significant differences were identified.

The CFA performed on the composition of archaeobotanical assemblages allowed to identify variations associated above all with the chronology of the sites (Chi^2^ = 304, df = 66, p<0.0001, total inertia = 1.63). It is however difficult to disentangle the effects of the variables related to site characteristics. Axis 1 (39.76% of variance) separates mostly cave and rock-shelter sites from the early Cardial/Epicardial period associated to wild plants, fruits and weeds, from the other sites associated to cereal remains (Fig 6A). Axis 2 (23.17% of variance) separates, in its upper part, Impressa sites associated to glume wheats and chaff, from late Cardial/Epicardial sites associated to free-threshing cereals in the negative part. The chronological trend on axis 2 is particularly obvious (Fig 6B). The calculation of a Kruskal-Wallis test confirm the significant difference between chronological phases (K = 8.65, df = 2, p = 0.013). However a significant difference can also be detected between sieving methods on axis 2 with a Mann-Whitney test (U = 5, p = 0.048). Because there is a clear progressive chronological pattern involving the three phases and because the organization of sites on axis 2 is for a large part determined by large items (caryopses of glume wheats vs. caryopses of free-threshing cereals), not much affected by the sieving method, we consider that overall changes in the archaeobotanical data are above all related to the chronology of the sites. No other significant pattern on axes 1 and 2 can be confirmed by the calculations of statistical tests.

However, it seems interesting to underline that, in most of the cave/rock-shelter sites from the early Cardial/Epicardial phase, wild fruits and herbaceous plants (possible field weeds) constitute about 50% or more of the plant remains.

### 3.2. Crops

**Fig 7:**
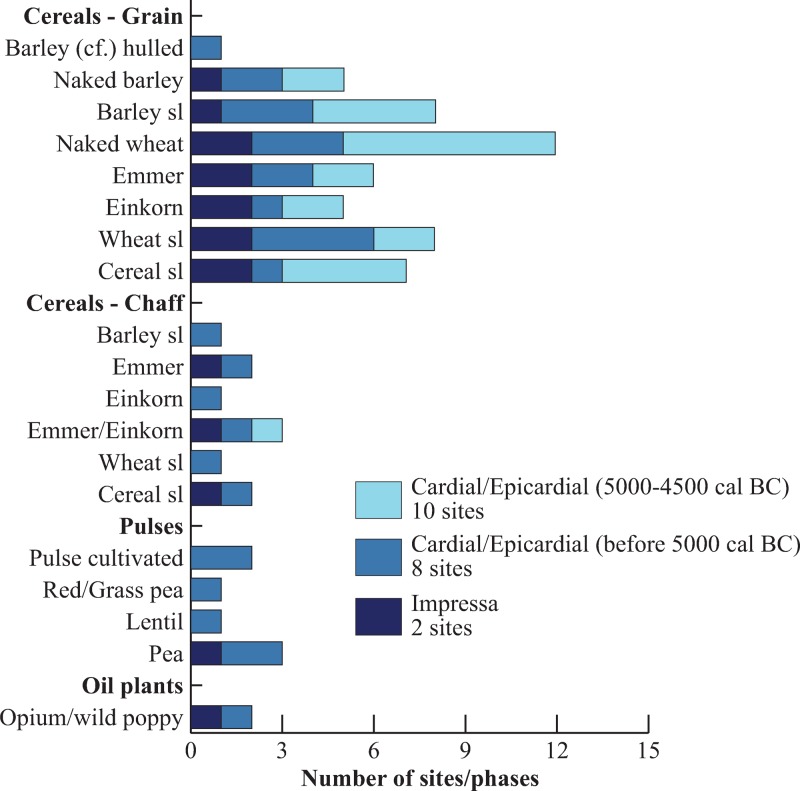
Frequency of the cultivated plants in the sites according to chrono-cultural phases.

Cereals are by far the most common crops. This is obvious according to both the percentage of plant remains (Fig 5) and the frequency of species in the different sites (Fig 7). The identified taxa are naked wheat (*Triticum aestivum/turgidum*), emmer (*T*. *dicoccum*), einkorn (*T*. *monococcum*), naked barley (*Hordeum vulgare* var. *nudum*) and, possibly, hulled barley (*H*. *vulgare* var. *vulgare*). In assemblages composed of many caryopses the twisted type of grain is always dominant over the straight type. This imply that barley belongs to the six-row type but a minor presence of two-row barley cannot be ruled out. Chaff remains are very scarce compared to grain, and as a matter of course they are only found in sites where fine sieving was performed. With the exception of two barley rachis segments (*Hordeum* sp.) from Taï chaff remains are entirely composed of glume bases of hulled wheats.

**Fig 8:**
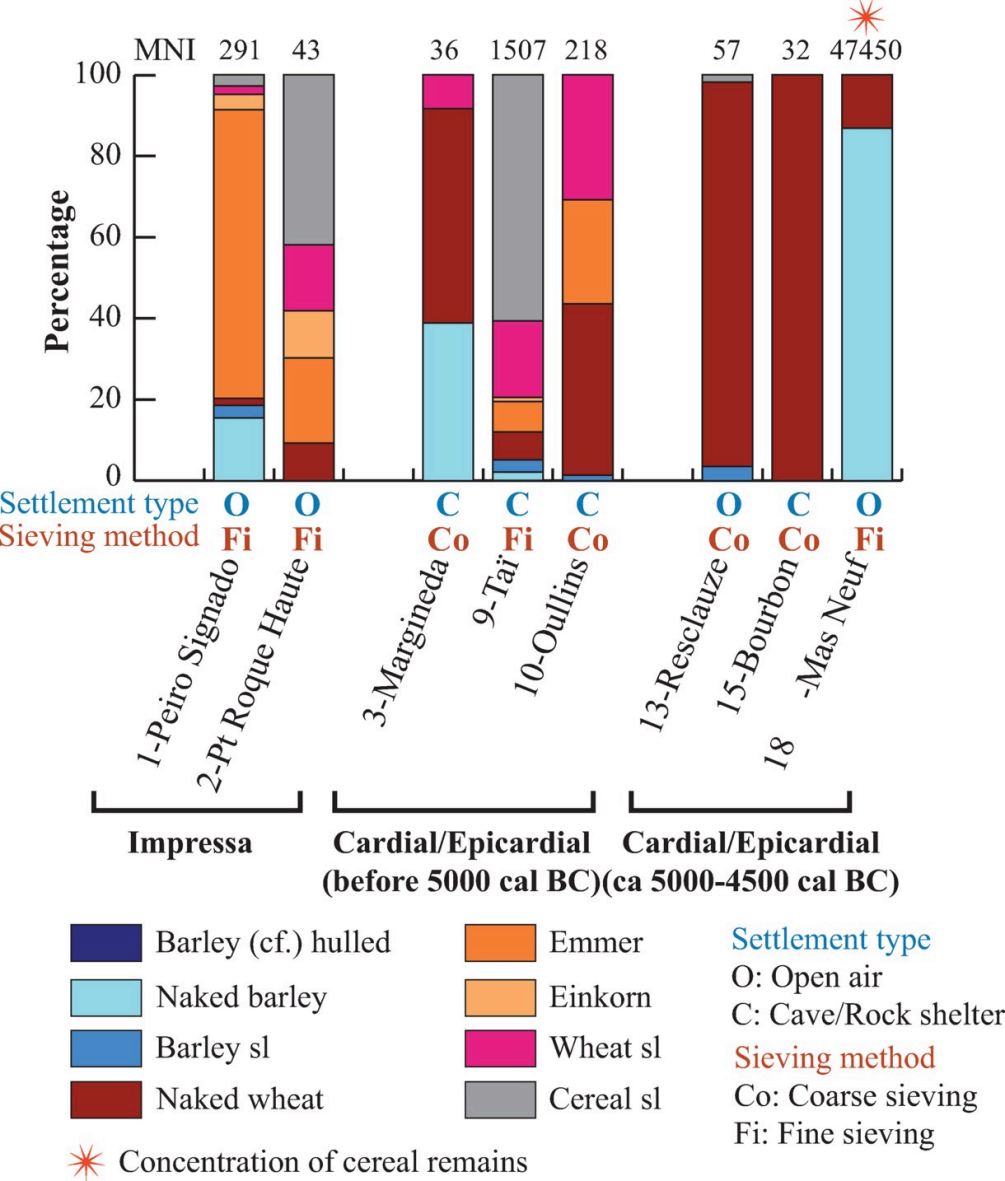
Proportions of the cereals in the sites (site/phases) expressed as percentages calculated on the MNI of cereal grains. The MNI is indicated on the graph at the top of the bar of each site/phase. Only the site/phases with MNI of cereal grains ≥ 30 are considered in this graph.

Except in Mas Neuf, barley is always outnumbered by wheat, according to the proportions calculated based on grain remains (Fig 8). As mentioned earlier, at Mas Neuf one single large concentration of barley was found in one of the sampled structures. Large grain-rich assemblages of this kind should be regarded as isolated accidents [eg. 26] and cannot therefore be considered as representative of the overall site economy. From a general point of view, naked wheat is the main type of wheat represented and also the main cereal in the Early Neolithic sites. It is the one found in the largest number of sites. In the sites where MNI of cereal remains is ≥30, naked wheat is by far the most common wheat during the second phase of Cardial/Epicardial, and most likely the first cereal during the whole Cardial/Epicardial period.

The earliest radiocarbon date for a caryopsis of naked wheat goes back to 5304–5066 cal BC (Baume d’Oullins, Figs 1 and 2). It is only in the two Impressa sites that naked wheat is clearly outnumbered by glume wheats, especially by emmer. Emmer is the most abundant glume wheat species in our sites. Emmer and einkorn are recorded during all periods but their abundance seems to decrease over time (Fig 8). The very early cultivation of emmer is confirmed by several radiocarbon AMS dates of grains recovered from the Impressa sites, all around 5850–5700 cal BC (Figs 1 and 2).

Barley is frequent at all periods (Fig 7) and well represented at two sites (Fig 8). This suggests that barley should be considered as a staple crop. The earliest radiocarbon date is recorded at Taï (direct dating: 5227–5041 cal BC) but barley grains have also been found at Impressa sites. Naked barley is the main form of barley found and possibly the only one to be actually cultivated during the entire Early Neolithic. Only one grain from Taï has been tentatively identified as hulled barley. This is not enough to assess that hulled barley was cultivated at that time in the area.

Pulses are more rarely found than cereals. Three domesticated species have been recorded: red/grass pea (*Lathyrus cicera/sativus*), lentil (*Lens culinaris*) and pea (*Pisum sativum*). The last named species is the most frequently found in the sites (Fig 7). No pulses have been detected so far during the second Cardial phase. Pulses are usually outnumbered by cereal remains at all the sites, except in Balma de l’Abeurador, but the total number of crop remains is too low at the site to assess correctly the relative proportion of cereals and pulses.

The only evidence for the cultivation of oil plants regards seeds of poppy (*Papaver setigerum/somniferum)* found at two sites. One single carbonized seed was identified in the Impressa site of Peiro Signado (ca. 5850–5600 cal BC) and 15 seeds in the Epicardial level of Taï (ca. 5200 cal BC). Unfortunately, it was not possible to identify the seeds either as the domesticated form (*P*. *somniferum* subsp. *somniferum*) or as its wild ancestor (*P*. *somniferum* subsp. *setigerum*).

### 3.3. Fruits

Fruit remains are common and diversified. At least 14 taxa are identified (S4 Table). They all consist of wild trees and shrubs, typical of the open Mediterranean evergreen oak forests (*Arbutus unedo–*Strawberry tree, *Juniperus communis/oxycedrus*—Juniper, *Pistacia lentiscus*- Mastic tree, *P*. *terebinthus*—Terebinth) and of more mesophilous conditions (*Cornus sanguinea*—Dogwood, *Corylus avellana*, *Prunus spinose*—Blackthorn, *Sambucus nigra/racemosa–*Black/Red elder, *S*. *ebulus–*Dwarf elder, *Vitis vinifera*—Grapevine). Unsurprisingly, the Mediterranean taxa are to be found only in sites located in areas under Mediterranean bioclimatic conditions. The presence of *Pistacia terebinthus* at Balma Margineda, 970 m asl in the Pyrenees, can be explained by the dry climatic conditions. This species, along with other typical components of the Mediterranean green oak forests, still grows today in the low Andorran valleys, [27].

Fruit remains of mesophilous taxa are recorded in sites located in the temperate but also in the Mediterranean areas, where they grow especially in the alluvial plains with deeper and moister soils.

**Fig 9:**
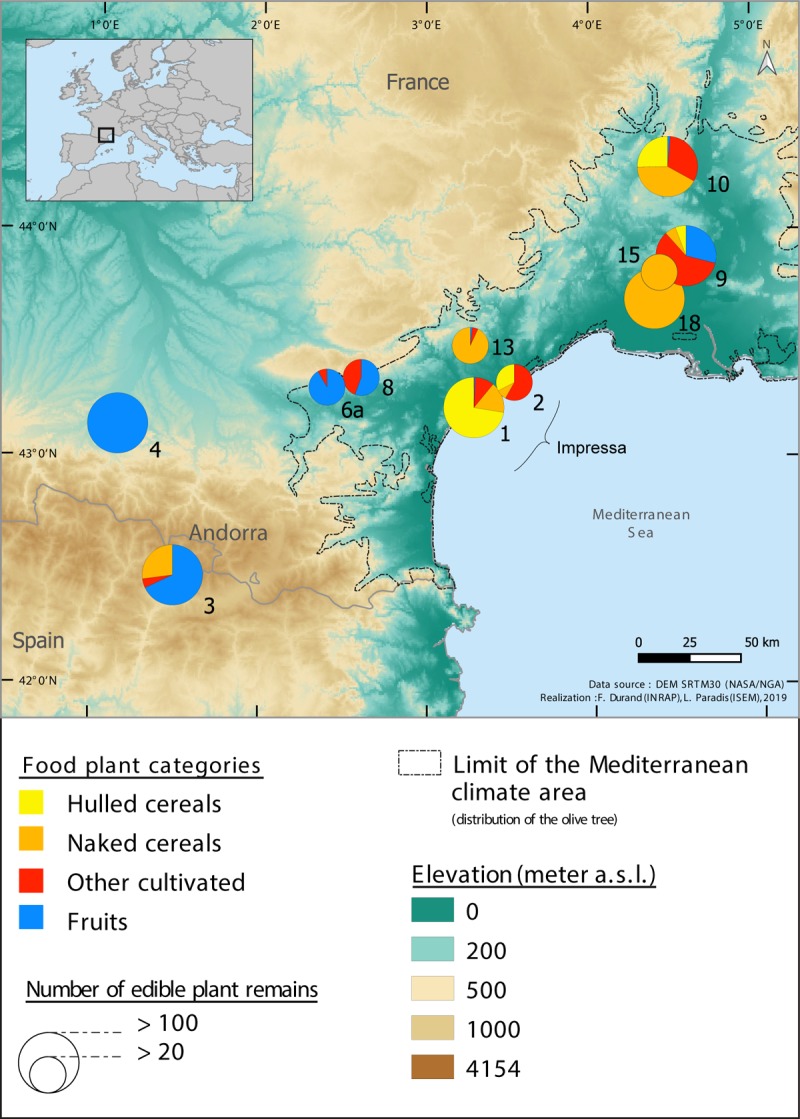
Composition of the archaeobotanical assemblages (% data) considering the main food plants: Hulled cereals, naked cereals, other cultivated (unidentified cereals, pulses and oil plants) and fruits.

The most frequent taxa in the archaeobotanical record are *Cornus sanguinea*, *Corylus avellana*, *Quercus* sp. and *Vitis vinifera*. In certain sites, the number of fruits remains is more important than that of cultivated plants. This is especially true for most of the caves and rock-shelters dating from the first part of the Cardial/Epicardial (Fig 5). Taï is the site where the highest number of fruit taxa is identified (Fig 9 and S5 Table). Several fruit taxa are only present at Taï and this is due for some part to the extensive sampling carried out at this site.

### 3.4. Wild herbaceous plants

The list of the weeds (wild herbaceous plants) include at least 40 taxa. The presence of these plants at the sites is generally low. Few sites, mostly caves and rock-shelters of the first Cardial/Epicardial phase (before 5000 cal BC), delivered more than 10 weed seeds (S5 Table).

The proportion of weeds was higher than 10% of the total assemblage at only four sites of this group, and somewhat surprisingly at two of these sites only coarse-sieving was carried out (Fig 5). Taï can again be seen as an exception, as the taxonomic spectrum includes at least 33 weed taxa, most of them being only recorded there. Most of the weed taxa are only recorded in one or two site/phases. Very few taxa are represented by more than 10 seeds: *Carex* sp.—sedge, *Chenopodium album*—goosefoot, *C*. gp *polyspermum–*manyseed goosefoot, *Hyoscyamus albus/niger*—henbane, *Linum* cf. *strictum–*upright flax, Poaceae—Grass, *Rumex* sp.—Dock and *Verbascum* sp.–Mullein.

From an ecological point of view, a large part of the taxa belong to synanthropic plant communities, typical of soils disturbed by human activities. Most of them are ruderal plants, encountered mainly, either on cultivated soils or in fallow land and waste places. Plants from these groups are reported at various sites. Segetal weeds (Secalinetea) are poorly documented and only recorded in Taï. Several taxa can be allocated to grasslands or dry pastures. However, most of them are identified with poor accuracy and therefore it is impossible to assign ecological significance with any certainty. Finally, various plants mostly thrive on wet grounds. They are encountered especially at Taï, where their presence could be associated with the banks of the Gardon River.

## 4. Discussion

### 4.1. The first crops of the northwestern Mediterranean: A farming system transposed from Italy

The sites of the Impressa cultural complex represent the oldest Neolithic settlements in the western Mediterranean, occupied around 5850–5650 cal BC [11, 28]. Few Impressa sites are known in Southern France, all of them located in the littoral area. Peiro Signado and Pont de Roque-Haute are considered as small settlements of populations originating from the Italian peninsula [11]. The available archaeobotanical assemblages [29] are clearly dominated by cereal remains, especially by hulled wheats (greater abundance of emmer than einkorn) (Fig 9). The minor presence of naked wheat is also recorded. Other cultivated plants, naked barley and common pea, have been found at Peiro Signado only. This could be explained by the greater number of samples and plant remains investigated in this site The early introduction of crops in Southern France is confirmed by the results of radiocarbon dating directly on cereal caryopses(ca. 5850–5650 cal BC).

At Peiro Signado crop processing activities are documented by the presence of dehusking by-products (emmer glumes). It seems highly probable that these Impressa colonists grew their own crops and can therefore be considered as the first farming communities in Southern France, occupying the same settlements during long-time periods; at least 80 years in Pont de Roque Haute and 140 years in Peiro Signado [14]. This is confirmed by the presence of blades typical of curved sickles, of numerous millstones, the abundance of locally produced pottery and the preparation and consumption of sheep [28, 30]. At Pont de Roque Haute, the discovery of a storage pit suggests long-term storage of food-plant products [8].

The focus on hulled wheats agrees with what is observed in more eastern sites of the Impressa complex. In the Pendimoun rock-shelter (close to the Italian border) the cereal remains recovered from the Impressa deposits (5730–5430 cal BC) are composed of naked barley, emmer and einkorn [31]. Emmer, einkorn and barley are also the dominant cereals in most of the early Neolithic Italian sites, including Coppa Nevigata, Masseria Santa Tecchia, Scamuso, Torre Sabea, Rendina (Puglia and Basilicata), Acconia and Catanzaro (Calabria), La Marmotta (Lazio), San Marco di Gubbio (Umbria) and Cava Barbieri (Tuscany) [32, 33, 34, 35, 36, 37]. The restricted number of fruit remains is another common pattern between Pont de Roque Haute, Peiro Signado and the Early Neolithic sites from Italy. Only *Pistacia* sp. and *Vitis vinifera* are sporadically recorded in Peiro Signado. Fruits are rare in the Italian sites with the exception of San Marco (Gubbio, Umbria). In Pendimoun, however, abundant remains of acorns (*Quercus* sp.) were found at the end of the Impressa occupation [31]. In short, it seems that the subsistence economy in the Impressa complex was globally focused on crops, including only a small contribution of gathered wild plants. A similar trend is recorded by archaeozoology. The majority of the Impressa sites, including those of the Languedoc, rely mostly on animal husbandry, especially sheep. A minor proportion of hunted animals is recorded [38, 39].

The homogeneity of the crop “package” and farming economy between the Languedoc sites and the Italian Impressa area can be explained by the speed of the colonization process. It would have involved rapid long-distance travel of small human groups by sea, resulting in a limited transformation of the technical and economic traditions [40, 41]. The time lapse between the emergence of the Impressa complex in Apulia and Calabria and the first settlements in Provence and Languedoc may have been as short as a century [42].

On the other hand, minor differences exist in the archaeobotanical spectra across the whole Impressa area. While naked barley is the only form of this cereal registered in the Languedoc, hulled six row barley is dominant in the Italian sites. Naked barley is apparently very rare, even unknown from the sites of Apulia. Elsewhere, it was recorded in Rendina and Lago di Rendina [43], in Basilicata, San Marco (Gubbio, Umbria), San Sebastiano di Perti (Finale Ligure, Liguria) [44] and in Pendimoun. Two row barley is identified at several Italian sites, especially in Apulia [35]. As in the French Impressa sites, naked wheat is regularly recorded in Italy, but is usually outnumbered by hulled wheats. Naked wheat is especially scarce in southern Italy, but more frequent towards central and northeastern Italy, as suggested by data from San Marco (Gubbio, Umbria), Cava Barbieri (Pienza, Tuscany), la Marmotta (lake of Bracciano, Lazio) [37] and Arene Candide (Finale Ligure, Liguria) [44]. However, many of these contexts are more recent than the oldest Impressa occupations in Southern Italy. In Apulia, naked forms are better represented after 5600 cal BC [35]. For the moment, it is difficult to understand the chronological or geographical trends in the rise of naked cereals. Concerning pulses, they are sporadically represented everywhere but seem more frequent in Southern than in Northern Italy [43].

Slight differences in agricultural practices, towards an increased role of naked cereals and a somewhat reduced contribution of pulses, can eventually be perceived over the whole Impressa area, from southern Italy to the Languedoc. It would however be necessary to investigate more thoroughly the perceived differences. The data available is still too limited. From a geographical point of view, a large part of the Italian results comes from Puglia and Basilicata, in the South, while archaeobotanical data are more limited in the other regions of Italy. In addition, a detailed chronological record is still missing. Not all the sites were occupied simultaneously and several of them were used for a large part of the 6^th^ millennium cal BC.

### 4.2. The predominance of naked cereals in the Cardial/Epicardial complex: Homogeneity and variability

A few centuries after the Impressa, the situation changed dramatically in southern France with the Cardial/Epicardial complex as naked cereals, especially naked wheat, became predominant in the archaeobotanical assemblages. This trend had already been noticed in previous works [45, 46].

Emmer is relatively important in two sites only, Taï and Baume d’Oullins, both located close to the Rhône valley and occupied before 5000 cal BC. Taï actually belongs to the Epicardial group while Baume d’Oullins is a typical Cardial site, which suggests that the use of hulled wheats is not restricted to any of these cultural groups. Emmer and einkorn are still recorded in certain sites between 5000 and 4500 cal BC but only by sporadic grains. According to the available data, the role of hulled wheat sharply declined after the Impressa phase and went on dwindling during the 5^th^ millennium. This fits well with what happens next, as during the first part of the Middle Neolithic (ca. 4500–4000 cal BC) naked cereals were largely predominant in Southern France [47].

Also, we have to consider that the role of hulled wheats could have varied spatially, as it seems to occur in Spain. In the Spanish Cardial/Epicardial the main cereals were also naked wheat and barley [48] but hulled wheats had a variable contribution from site to site; they occasionally played an important role in several areas of the country, especially during the 6th millennium cal BC. This was the case in the earliest Neolithic sites of the Pays Valenciano [48], in areas of the Catalan coast [49], in Central Spain [50] and in the Basque country [51]. In Northern Italy some contrasts are also noticed, with free-threshing wheats important in the central and North-Western parts while rare in the North-East [52].

In short, data available in Southern France for the period ca 5500–4500 cal BC fit well in the wider picture of the North-Western Mediterranean; agriculture was dominated by naked cereals, especially free-threshing wheats, but also included significant contributions of hulled cereals in certain areas. Their role seems to have decreased through time. So the shift from hulled to naked wheats is the prominent feature of agricultural change during the Early Neolithic. In France, this shift seems all the more sharp due to the chronological gap between the Impressa and the first Cardial sites [14]. It is possible that the sites that could eventually document a gradual decline in glume wheats are still to be discovered.

Shifts between hulled and naked cereals occurred at different times and places in the history of agriculture and can be explained by multiple, possibly intertwined factors. Cereal species differ according to their growth requirements, to the technology required for cultivation and processing, to possible uses of grain and straw and to socio-cultural preferences [53]. The main difference between hulled and naked cereals is that the naked forms are more easily processed. With naked cereals, glumes are simply removed by threshing and no labor consuming dehusking is required in order to obtain clean grain. This is especially appreciable when the grain is dedicated to human food. In recent times, naked barleys were usually favored in places where they contributed significantly to human diet [54]. The priority given to naked cereals should then be regarded first as a human choice. But this choice could have been helped by more favorable climate conditions in the North-Western Mediterranean compared with those of Southern Italy at the beginning of the Impressa period. Hulled wheats and barley are regarded as hardier and more tolerant to harsh environmental conditions compared to naked cereals, especially because of the protection offered by the hulls from insects and diseases [54, 55]. We don’t know which kind of naked wheat was cultivated in South-Western France. Tetraploid (*Triticum durum* and *T*. *turgidum*) and hexaploid (*T*. *aestivum*) naked wheats can be discriminated in archaeobotany only from rachis remains, but none have been found in the area. In Northern Italy and Spain both naked wheat types are identified but, in the sites where waterlogged conditions allow for a good preservation of chaff, tetraploid naked wheat is either clearly predominant (la Draga, lake of Banyoles, Catalunya) [56] or the only naked wheat recorded (la Marmotta, lake of Bracciano, Lazio) [37]. We should therefore consider that in Southern France naked wheat was also mainly of the tetraploid type. Tetraploid naked wheats are better adapted to dry Mediterranean climates than hexaploid forms [57].

Climate reconstructions show that during the whole 6^th^ cal BC millennium the North-Western Mediterranean coast was characterized by dry summers, while wetter and cooler conditions than those of today are registered in Southern Italy [58, 59]. Additionally, the climate of the Northern Mediterranean would be characterized by strong seasonal contrasts during the second part of the 6^th^ millennium cal BC, with heavy rain from autumn to spring, and dry summers [60]. These warmer and drier conditions were certainly more favorable to naked cereals than those of the mid-6^th^ millennium cal BC in South Italy. In Apulia, the better representation of naked cereals during the second half of the 6^th^ millennium cal BC is attributed to the tendency towards drier climate conditions [35].

Evidence for other cultivated plants in addition to cereals in the Cardial/Epicardial agriculture of Southern France is rather scarce. Pulses include red/grass pea (*Lathyrus cicera/sativus*), lentil (*Lens culinaris*) and with greater frequency pea (*Pisum sativum*).

The scarcity of pulses is a common feature in the whole North-Western Mediterranean. However, in some specific areas or in some particular sites, pulse seeds of different species can be present in abundance (dozens of seeds or more). During the Early Neolithic, pulses were slightly more important towards the South, in Southern Italy [43] and in Southern Spain [53] but the example of Sammardenchia-Cueis, Udine, shows that pulses can be diversified and abundant in Northern Italy, outside the Cardial area [37].

Concerning oil plants in the Early Neolithic of the western Mediterranean, they are represented by rare finds of flax (*Linum usitatissimum*) and opium poppy (*Papaver somniferum*). Both species are reported in Italy [37, 43] and Spain [48, 56, 61].

Flax has never been recorded in Southern France but this could change in the future with more extensive sampling and fine sieving. On the other hand, several seeds of opium poppy (*Papaver somniferum*) have been found in Taï (ca. 5200 cal BC) and, due to their location in one single pit, they are regarded as cultivated rather than wild plants [19]. A single *Papaver somniferum* subsp. *setigerum/somniferum* seed was also found at the Impressa site of Peiro Signado but this is not enough to argue in favor of an early cultivation of opium poppy. The wild ancestor of opium poppy (*P*. *somniferum* subsp. *setigerum*) is actually regarded as native to the western Mediterranean. It was consequently domesticated here rather than imported from the East as other Neolithic cultivated plants [62].

The charred seed recovered in Peiro Signado suggests that man was already familiar with the plant, whether poppy was already cultivated or simply growing as a weed in cultivated fields and involuntarily collected with the harvest.

According to the latest overview, opium poppy was identified in several Early Neolithic sites in the Western Mediterranean at least from 5300 cal BC onwards [63]. In Spain, it is reported at Cueva de los Murciélagos de Zuheros (Córdoba Province, Andalucia), at sites located in the Meseta and at la Draga, in Catalunya [48, 56, 64]. In Italy, the recovery of numerous opium poppy remains at la Marmotta, favored by the waterlogged conditions, could even be more ancient than 5300 cal BC [37]. From ca 5200 cal BC onwards, poppy became common in the Linearbandkeramik area [65], most probably due to early contacts with the Cardial/Epicardial areas.

### 4.3. Consumption of fruits and wild resources

The consumption of wild plant foods and the persistence of gathering activities during the Neolithic period cannot be easily traced, mostly due to taphonomic issues. As a result, the contribution of wild plant foods to human diet is largely underestimated in many Neolithic sites, especially in dry settlements where only carbonized remains are preserved [66, 67]. Collected foodstuffs eaten outside the settlements are invisible. Leaves, shoots, roots and tubers rarely leave any trace in the carbonized records [68]. It is also very difficult to distinguish the seeds of herbaceous plants which may have been carried to the settlements for their food value, from those brought for the animals or just by accident with the crops or for any other reason.

Due to their usually high nutritional value, and to the fact that they are less likely to be transported unintentionally to the settlements than weeds, fruits from trees and shrubs are regarded in most archaeobotanical studies as the most straightforward evidence of the gathering of wild plant foods. The study of a waterlogged site such as la Draga nevertheless shows how carbonized items represent merely a fraction of the fruit record and, consequently, how fruit consumption is underestimated at ‘dry’ Early Neolithic sites [56, 69].

In the Early Neolithic sites of Southern France, fruits remains are frequently recorded. They even occasionally outnumber cultivated plant remains. The most frequent are dogwood fruits, grapes, hazelnuts and acorns. The contribution of fruits is higher in cave and rock-shelters of the hinterland, which mostly date from the first Cardial/Epicardial phase. Fruits are more rarely found at the Impressa and late Cardial/Epicardial sites of the lowlands (Fig 9). Of course the available data is still too limited to be representative but this pattern is consistent with the situation observed by archaeozoology. In the area, the contribution of hunted animals to the economy generally increases with time, higher in Cardial than in Impressa sites [38, 39]. Furthermore, a spatial trend also exists: hunting activities are more important in hinterland than in littoral sites [38, 39]. Our pattern equally reminds us of what is observed on the other side of the Pyrenees, in Spain, where charred fruit remains are more common in mountain sites. This is interpreted as the result of particular fruit processing activities taking place at these settlements [67, 69]. Hazelnuts and acorns especially, recovered in large amounts, would have been roasted in bulk to facilitate their storage. On the other hand, other scholars argue that it is the day-to-day consumption of hazelnuts that may favor their exposure to fire, by discarding the unwanted shells into domestic fires or even using them as fuel [70]. In our case the data are insufficient to favor any of these two hypotheses. No concentration of fruit remains, or any other possible indication of specialized fruit processing activities, have been found up to now.

Another practice which may well explain the better representation of fruits in certain caves and rock-shelters is the collection of leaf-fodder for domestic animals. In Spain, Pérez-Jordà *et al*. [48] considers that remains of wild fruits are more common in the caves related to animal husbandry than in others. In Taï, where the largest number and diversity of fruit remains was found, we have argued that the strong representation of wild plants could result from the collection of fodder or litter for animals kept at the site [19].

In summary, the better representation of charred fruits in caves and rock-shelters from the hinterland is likely due to several economic activities, not necessarily exclusive of each other (importance of fruits in the diet, specific fruit processing, and stabling of animals). It is impossible to say to what extent the importance of the fruits is related to the nature of the sites, in cave or rock-shelters, or to their location in the hinterland.

### 4.4. Cultivation techniques in the Cardial complex and the diffusion of agriculture

It is also very difficult to estimate the importance of local agricultural production and the variability between sites, especially from the littoral to the hinterland. Typical by-products of crop processing, chaff remains and weed seeds, are rare in our sites, even in the fine sieved samples. The most common chaff remains, glume bases of hulled wheats, are always outnumbered by hulled wheat grains in the assemblages. The highest numbers of weed seeds are found in caves and rock-shelters, some probably brought by or for domestic animals (faeces, fodder) rather than as the result of crop processing activities.

The scarcity of chaff and weed seeds could result from several reasons [e.g. 70, 71], especially crop processing away from the settlements or processing in bulk immediately after the harvest. In such cases by-products have less chance to be carbonized in the domestic hearths. Post-harvest processing in bulk is especially likely concerning naked cereals–dominant during the Cardial/Epicardial- but is also possible for hulled wheats, even if not as straightforward because the removal of the glumes require time-consuming dehusking [72]. Processing in bulk has previously been hypothesized in Languedoc for the Late Neolithic [73].

The scarcity of weed seeds could be favored by the harvesting of cereals high on the straw, leaving most of the weeds in the field. The only probable residue of storage that has been found in Mas Neuf contained no weed seeds at all. This could result either from harvesting only the cereal spikes or from a thorough cleaning prior to storage. High harvest has also been suggested in the case of Early Neolithic La Draga, in Catalunya [56, 74]. This method is in agreement with the harvesting tools found in Southern France. From ca 5300 cal BC onwards, the reaping knives with parallel-hafted blades recorded are better suited to harvest the ears only [75, 76].

Even if scarce, most of the herbaceous taxa identified in our sites belong to synanthropic plant communities, growing on cultivated and ruderal land, which suggests the existence of cultivated fields nearby. This is especially evident at Taï, where the dominance and diversity of segetal and ruderal weeds can be considered as evidence of local cultivation. The information provided by weeds is nevertheless insufficient to trace the spread of farming practices in South-Western France.

However, in the Mediterranean lowlands, farming activities are recorded at Cardial and Epicardial sites based on the presence of dedicated structures and tools. Underground storage pits have been found in several sites (Mas de Vignoles 10, Mas Neuf, Taï, Aspre del Paradis). The only large concentration of grain was recorded, as a secondary deposit, in one of these pits, in Mas Neuf, as mentioned before. It is believed that high density grain assemblages are more likely to occur at sites where cereals are produced and consumed on a large scale [26]. Reaping knives (Taï, Mas de Vignoles 10as well as grinding tools (Mas de Vignoles 10, Mas Neuf, Taï, Aspre del Paradis) have also been found.

Towards the hinterland, the Early Neolithic sites become more scattered, with poorly characterized settlements, often located in caves and rock-shelters. Consequently, the evidence of cultivation activities is very poor. In certain sites it has so far been impossible to recover any reliable archaeobotanical data. In others (Buholoup, Roc de Dourgne) only wild plant remains were identified. However, at Balma Margineda (Andorra) and Baume d’Oullins (Gard), cereal grain was clearly used. In both cases the radiocarbon dating of wheat and barley grains confirmed their chronology.

In all likelihood, agriculture was practiced in the hinterland but its role remains extremely difficult to assess and is obviously underestimated. It must be reminded that even in Neolithic sites dependent on cereals, grains are usually relatively rare in the carbonized record, and can be underrepresented in relation to fruit remains such as hazelnut shells [70]. Pollen anthropogenic indicators bear evidence of agro-pastoral activities in the hinterland, but the signal is variable in space and occurs later than in the littoral area, about 5300–4500 cal BC in the Pyrenean foothills, and during the second half of the Early Neolithic period at higher altitudes [77, 78]. Animal husbandry was carried out in the whole area during the Early Neolithic, despite the evidence of the importance of wild fauna in the hinterland [38]. In the Southern Pyrenees, not far from Balma Margineda, the existence of local farming activities is confirmed in Coro Trasito and Camp del Colomer by the presence of cereal grain and chaff, remains of millstones and storage pits [69, 79, 80]. In the El Trocs rock-shelter however, the absence of crop-processing by-products would suggest that fully processed wheat and barley grain was transported to the site from elsewhere [81]. The diversity of situations must have been as important in the Southern Pyrenees as in South-Western France. It is thought that the dispersion of small size Cardial settlements in all biotopes made it possible to exploit the greatest diversity of resources and to promote economic complementarities between sites and territories through seasonal movements or exchanges [9, 41]. The role of agriculture in these networks is still very poorly known. These questions represent fundamental issues for further research.

## Conclusions

The main objective of this article was to provide a first attempt to characterize food plant resources, cultivation practices and their variations in time and space during the Early Neolithic in South-Western France, based on a critical review of the current archaeobotanical data. In the context of the Neolithization of the Western Mediterranean, carried out by human communities of the Impressed Ware complex, South-Western France is an interesting area, as it offers the opportunity to investigate 1) the chrono-cultural transition from Impressa to Cardial/Epicardial groups, the two main cultural communities responsible of this Neolithization, 2) the diffusion of agricultural economy from the Mediterranean littoral area to the hinterland.

It proved however difficult to acquire new data from recent excavations on Early Neolithic sites, as these, regularly limited to few and poorly characteristic archaeological features, often go undetected. Large scale excavations are consequently rare. Up to now, archaeobotanical sampling carried out at new excavations has proven negative in several sites. On the other hand, the example of Taï shows that, in certain cases, intensive field-work gives the opportunity to perform extensive sampling of well-preserved carbonized archaeobotanical material.

It becomes evident that archaeobotanical data from early studies still have a major part to play in the available documentation. The critical revision of all the data from early studies provides a clean and validated dataset. It is however insufficiently representative from many points of view: restricted sampling, inadequate recovery techniques at many sites, limited number of sites spread over a large area and uneven spatial distribution. As a result very few datasets can be considered outside the Mediterranean region. This illustrates first the difficulty to excavate and sample secure Early Neolithic stratigraphic contexts in the temperate zone.

From a global point of view, the results from South-Western France fit and complement satisfactorily the information from Italy and Spain. The farming economy in the Impressa sites of Languedoc focusses on hulled wheats, in agreement with the situation observed in Italy. The minor contribution of naked cereals identified in the Impressa of Languedoc could be an innovation compared to the oldest sites in Southern Italy. Naked cereals later play a dominant role in the Cardial/Epicardial agriculture in France and Spain, but hulled cereals still are of some importance locally, especially during the 6^th^ millennium cal BC. This variability and the dynamics of the shift from hulled to naked cereals still require further detailed investigation, but this will only be possible with a systematic and careful re-examination of the existing documentation at a macro-regional scale, in order to have a certified and chronologically detailed archaeobotanical record all-over the Western Mediterranean.

Within the French Cardial/Epicardial area, our data suggest a more important role of wild fruits in hinterland sites. Concurrently these sites show a more tenuous contribution of crops and agriculture. Comparisons can be made with the situation in neighboring regions or with results from faunal remains. However, in the present state of knowledge, it would be far too simplistic to draw any unequivocal conclusion about the weak role of agriculture in the temperate area. First of all, the poor evidence of crops and cultivation activities results from difficulties to identify and excavate sites and to perform large-scale archaeobotanical sampling. These difficulties are in turn caused by the small size and variability of the sites. Our hypothesis is that this variability illustrates both the diversity of activities and economic strategies implemented by Cardial/Epicardial communities in the temperate zone and the variable economic role of agriculture.

## Supporting information

S1 Table Newly investigated sites with assumed Early Neolithic occupations. In brackets: Department number.
(XLSX)

S2 Table List of the current archaeobotanical data from sites and site/phases that have been retained after revision of stratigraphic, cultural and radiocarbon data. In brackets: Department number.
(XLSX)

S3 Table Radiocarbon dates performed on carbonized seeds. (XLSX)

S4 Table Archaeobotanical results related to cultivated plants and wild fruits synthesized according to the main chrono-cultural phases. (XLSX)

S5 Table Detailed archaeobotanical results for each Early Neolithic site. (XLSX)
